# The Impact of Total Variation Regularized Expectation Maximization Reconstruction on ^68^Ga-DOTA-TATE PET/CT Images in Patients With Neuroendocrine Tumor

**DOI:** 10.3389/fmed.2022.845806

**Published:** 2022-03-11

**Authors:** Lin Liu, Hanxiang Liu, Shijie Xu, Shumao Zhang, Yi Tao, Greta S. P. Mok, Yue Chen

**Affiliations:** ^1^Department of Nuclear Medicine, The Affiliated Hospital of Southwest Medical University, Luzhou, China; ^2^Nuclear Medicine and Molecular Imaging Key Laboratory of Sichuan Province, Luzhou, China; ^3^Academician (Expert) Workstation of Sichuan Province, Luzhou, China; ^4^United Imaging Healthcare, Shanghai, China; ^5^Biomedical Imaging Laboratory (BIG), Department of Electrical and Computer Engineering, Faculty of Science and Technology, University of Macau, Taipa, Macao SAR, China

**Keywords:** PET/CT, ^68^Ga-DOTA-TATE, TVREM, OSEM, image quality

## Abstract

**Objective:**

The aim of this study was to investigate the effects of the total variation regularized expectation maximization (TVREM) reconstruction on improving ^68^Ga-DOTA-TATE PET/CT images compared to the ordered subset expectation maximization (OSEM) reconstruction.

**Method:**

A total of 17 patients with neuroendocrine tumors who underwent clinical ^68^Ga-DOTA-TATE PET/CT were involved in this study retrospectively. The PET images were acquired with either 3 min-per-bed (min/bed) acquisition time and reconstructed with OSEM (2 iterations, 20 subsets, and a 3.2-mm Gaussian filter) and TVREM (seven penalization factors = 0.01, 0.07, 0.14, 0.21, 0.28, 0.35, and 0.42) for 2 and 3 min-per-bed (min/bed) acquisition time using list-mode. The SUV_mean_ of the liver, background variability (BV), signal-to-noise ratios (SNR), SUV_max_ of the lesions and tumor-to-background ratios (TBR) were measured. The mean percentage difference in the SNR and TBR between TVREM with difference penalization factors and OSEM was calculated. Qualitative image quality was evaluated by two experienced radiologists using a 5-point score scale (5-excellent, 1-poor).

**Results:**

In total, 63 lesions were analyzed in this study. The SUV_mean_ of the liver did not differ significantly between TVREM and OSEM. The BV of all TVREM groups was lower than OSEM groups (all *p* < 0.05), and the BV of TVREM 2 min/bed group with penalization factor of 0.21 was considered comparable to OSEM 3 min/bed group (*p* = 0.010 and 0.006). The SNR, SUV_max_ and TBR were higher for all TVREM groups compared to OSEM groups (all *p* < 0.05). The mean percentage difference in the SNR and TBR was larger for small lesions (<10 mm) than that for medium (≥10 mm but < 20 mm) and large lesions (≥20 mm). The highest image quality score was given to TVREM 2 min/bed group with penalization factor of 0.21 (3.77 ± 0.26) and TVREM 3 min/bed group with penalization factor of 0.35 (3.77 ± 0.26).

**Conclusion:**

TVREM could reduce image noise, improve the SNR, SUV_max_ and TBR of the lesions, and has the potential to preserves the image quality with shorter acquisition time.

## Introduction

Neuroendocrine tumors (NETs) are a heterogeneous group of cancers derived from the diffuse neuroendocrine system. Neuroendocrine cells are distributed in every organ, the primary NET may occur in any part of the human body, and therefore early diagnosis of NET is very important for treatment (1). NET's early diagnosis methods include multiple CT, MRI, and endoscopic ultrasound. However, the specificity and sensitivity of these methods to NET are not high. One of the characteristics of neuroendocrine tumors is the high expression of somatostatin receptors (SSTR) (2). SSTR-expression tends to correlate inversely with tumor grade and differentiation, and consequently, the role of SRI is more limited in high grade and particularly in poorly differentiated carcinomas (3). Different types of tumors, primary tumors at different locations, and tumors in different differentiation grades have different somatostatin receptors over-expressed. There are 5 somatostatin receptors (SSTR 1-5) widely expressed in both normal tissues and tumors. In most tumors, SST2 and 5 are usually over-expressed (4, 5). This expression enables imaging with high sensitivity using PET with radioactively labeled somatostatin analogs such as ^68^Ga-DOTA-TATE. PET with ^68^Ga-labeled somatostatin ligands is well-established as a tool for localizing the primary tumor in metastatic NET (6, 7).

The radionuclide, the performance of the equipment, and the reconstruction algorithm are important factors that affect the precise positioning of the lesion. Ordered subsets expectation maximization (OSEM) is the clinical standard reconstruction method. However, due to the special requirements for noise and reconstruction time in clinical applications, the iteration of OSEM must be stopped before converging. This always leads to underestimation of radio-tracer uptake and low contrast of the lesion. Bayesian penalized likelihood reconstruction (BPL) can reduce image noise and increase lesion contrast compared to OSEM reconstruction (8–10). Total variation regularized expectation maximization (TVREM), a new BPL algorithm, was introduced recently (HYPER Iterative, United Imaging Healthcare). Related studies have shown that TVREM algorithm in ^68^Ga-PSMA imaging can ensure image quality while shortening the acquisition time (11). Therefore, as a BPL algorithm, TVREM has the potential to further increase the detection rate of lesions, shorten the acquisition time or reduce the dose (reduce administrated dose) for ^68^Ga-DOTA-TATE PET imaging.

The purpose of this research is to explore the effect of TVREM algorithm on ^68^Ga-DOTA-TATE PET/CT image quality, and the possibility of shortening the acquisition time and reducing the radiation burden.

## Methods and Materials

### Study Population

Between March 1st, 2021 and May 30th, 2021, 17 patients with neuroendocrine tumors undergoing ^68^Ga-DOTA-TATE PET/CT were enrolled in this study retrospectively. The study was approved by the ethics committee of the Affiliated Hospital of Southwest Medical University and was in accordance with the 1964 Helsinki declaration and its later amendments. The informed consent of this retrospective study was waived.

### Preparation of ^68^Ga-DOTA-TATE

^68^Ga stock solution was made by rinsing the ^68^Ga generator using 4 mL of 0.05 mol/L hydrochloric acid. The pH value was adjusted to 3.5–4.0 with sodium acetate (0.25 mol/L). Twenty μg of DOTA-TATE (ABX, Germany) was added to the solution and mixed, which was then heated at 95°C for 10 min. The solution was first purified by a Sep-Pak C18 column, and then was filtered using a sterile filter to obtain ^68^Ga-DOTA-TATE injection after washing the column with 50% ethanol solution and saline solution. The radiochemical purity was >99% analyzed by a radioactive high performance liquid chromatography (Radio-HPLC).

### Image Acquisition

For ^68^Ga-DOTA-TATE PET/CT imaging, the activity of intravenously injected according to the patients' weight (2.04 ± 0.19 MBq/kg). PET/CT images were acquired after 50–70 min of intravenous administration using a digital PET/CT scanner (uMI780, United Imaging Healthcare). A non-enhanced CT was used for attenuation correction of PET and anatomic localization. The CT parameters were as follows: 120 kV, 100 mAs, rotation of 0.8 s, and 3-mm slice thickness. After CT scanning, a whole-body PET scan was performed with 5–6 bed positions and 3 min/bed.

Attenuation corrected images were reconstructed with OSEM and TVREM. All images were reconstructed with time of flight (TOF), point spread function model (PSF), 600 mm field of view (FOV), 128 × 128 matrix, and slice thickness 3.0 mm. The OSEM reconstruction was performed with 2 iterations, 20 subsets and 3.2 mm of full width at half maximum (FWHM). Reconstruction with TVREM was performed with seven penalization factors: 0.01, 0.07, 0.14, 0.21, 0.28, 0.35, and 0.42. List-mode data were re-binned using the first 2 min /bed of the data and reconstructed with the same parameters.

In total, there were 16 groups of PET images in the final evaluation. We named these groups as OSEM_2 and OSEM_3 for OSEM with 2 and 3 min/bed; R201, R207, R214, R221, R228, R235, R242 for TVREM with different factors (0.01–0.42 for 2 min/bed) and R301-R342 for TVREM with factors (0.01–0.42 for 3 min/bed).

### Clinical Studies Analysis

All the images were evaluated by an experienced nuclear medicine doctor with HERMES (HERMES, Stockholm, Sweden), and the regions of interest (ROI) were drawn on transaxial images around the tumor lesions to perform a semiquantitative analysis. A spherical (approximated 3 cm in diameter) in the right liver lobe was used as the activity background for each patient. The mean standardized uptake value (SUV_mean_) and the standard deviation (SD) of liver were recorded. Background variability (BV) was used as parameter to evaluate the image noise level and calculated as follow:


(1) $$\begin{array}{r} BV=SD/SUV_{mean} \end{array}$$

Maximum SUV (SUV_max_) values of lesions were recorded. Signal-to-noise ratios (SNR) and tumor-to-background ratios (TBR) were calculated as follows:


(2) $$SNR=(SUV_{\max}\text{ of lesion)}/BV$$

(3) $$TBR=(SUV_{\max}\text{ of lesion)}/(SUV_{mean}\text{ of liver)}$$

Lesions were also divided and compared according to their size (diameter <10 mm, ≥10 mm but <20 mm, and ≥20 mm). The diameter of lesions were calculated as follows:


(4) $$diameter_{lesion}=3\sqrt{(Volume_{lesion}/4\pi )\times 3}\times 2$$

where the volume of lesions was measured on PET imgaes.

### Clinical Evaluation

Two experienced nuclear physicians evaluated the image quality of ^68^Ga-DOTA-TATE PET images reconstructed using OSEM and TVREM independently on a commercial workstation (uWS-MI R004, United Imaging Healthcare). The nuclear physicians evaluated the images using a 5-point scale without knowing the reconstruction parameters (Table 1). The image of 5-point was with excellent image quality, almost free of noise, ideal contrast and sharp border. The image of 4-point was with good image quality, and its noise did not affect the identification and diagnosis of the lesion at all. The image of 3-point was with moderate image quality, obvious noise, and sufficient lesion delineation to make a diagnosis. The image of 2-point was barely acceptable, and the noise was large, which affected the diagnosis. The image of 1-point had the worst image quality and could not be used for diagnosis at all.

**Table 1:** The 5-point scale of image quality.

**Scores**	**Descriptions**
5	Excellent image quality, almost free of noise, ideal contrast and sharp border
4	Good image quality, and its noise did not affect the identification and diagnosis of the lesion at all
3	Moderate image quality, obvious noise, and sufficient lesion delineation to make a diagnosis
2	Barely acceptable, and the noise was large, which affected the diagnosis
1	The worst image quality and could not be used for diagnosis at all

### Statistical Analysis

SPSS 22.0 statistical analysis software (IBM, Armonk, NY, USA) was used for statistical analysis, and Graphpad8.0 was used for graphing. The data were presented as mean ± SD. The SUV of OSEM_3 was served as the reference for the comparison between different reconstruction groups. Shapiro-Wilk test were used to test the normal distribution of data. Paired samples were compared using the Wilcoxon signed-rank test and paired *t*-test. The inter-evaluator agreement was measured by Cohen's κ. A *p* < 0.05 was considered significant.

## Results

A total of 17 patients (7 women and 10 men; mean age ± SD, 48 ± 17 years) with suspicion and diagnosis of NET were enrolled and the presence of lesions was confirmed by CT, MRI, ^18^F-FDG PET and biochemical evidence of NETs. The characteristics of the study population were summarized in Table 2.

**Table 2:** Characteristics of study population.

**Patient number**	**Age (years)**	**Gender**	**Tumor location**	**Ki-67**	**NET grade**	**The number of lesions**
1	47	F	Pancreas NET	3%	G2	7
2	44	F	Adrenal pheochromocytoma	20%	G2	7
3	38	M	Adrenal pheochromocytoma	8%	G2	5
4	57	M	Pancreas NET	5%	G2	7
5	38	F	CBT	3%	G2	7
6	31	F	Pancreas NET	10%	G2	3
7	34	M	Pancreas NET	10%	G2	1
8	48	M	Pancreas NET	6%	G2	3
9	27	F	Pancreas NET	2%	G2	2
10	30	M	Pelvic NET	8%	G2	7
11	64	F	Pancreas NET	1%	G1	2
12	15	F	Hypophysoma	10%	G2	1
13	63	M	Pheochromocytoma	12%	G2	1
14	67	M	Highly differentiated neuroendocrine tumor of the left laryngeal wall	10%	G2	1
15	73	M	Mediastinal NET	80–90%	G3	2
16	66	M	Small cell NET of the left neck	40%	G3	4
17	75	M	Left groin NET	20%	G2	3

*NET, neuroendocrine tumor; CBT, carotid body tumor*.

### Lesion Analysis

In total, 63 lesions were identified in both reconstructions. The lesions' diameter ranged from 7.0 to 60.0 mm. The mean values of SUV_mean_ were not significant different between TVREM and OSEM (all *p* > 0.80). The mean values of BV in OSME_2 and OSEM_3 were 8.2 ± 1.9% and 7.2 ± 1.9%, the BV in TVREM groups decreased with the increase of penalization factors and the BV of R221 group was considered comparable to OSEM_3. The SUV_max_, SNR, and TBR were higher in all TVREM groups than OSEM (all *p* < 0.05). The SNR increased with the increase of penalization factor. Moreover, the SUV_max_ and TBR of the lesions decreased as the penalization factors increased (Table 3).

**Table 3:** SUV_mean_, background variability, SUV_max_, SNR and TBR of the clinical study.

	**Background SUV_**mean**_**	**Background variability %**	**SUV_**max**_**	**SNR**	**TBR**
OSEM_2	7.62	8%	19.06	268.20	2.62
R201	7.56	8%	20.82	281.92	2.88
R207	7.56	8%	20.78	294.46	2.87
R214	7.57	8%	20.73	309.71	2.86
R221	7.57	7%	20.68	325.63	2.85
R228	7.60	7%	20.63	341.02	2.85
R235	7.60	7%	20.57	357.25	2.84
R242	7.60	6%	20.53	373.98	2.83
OSEM_3	7.56	7%	18.94	297.89	2.61
R301	7.61	8%	20.98	299.67	2.89
R307	7.61	8%	20.95	305.71	2.89
R314	7.58	7%	20.92	317.58	2.88
R321	7.58	7%	20.89	329.07	2.88
R328	7.62	7%	20.90	339.40	2.88
R335	7.61	7%	20.85	352.69	2.88
R342	7.61	6%	20.81	364.06	2.87

*SUV, standardized uptake value; SNR, signal-to-noise ratio; TBR, tumor-to-background ratio*.

**Figure 1:**
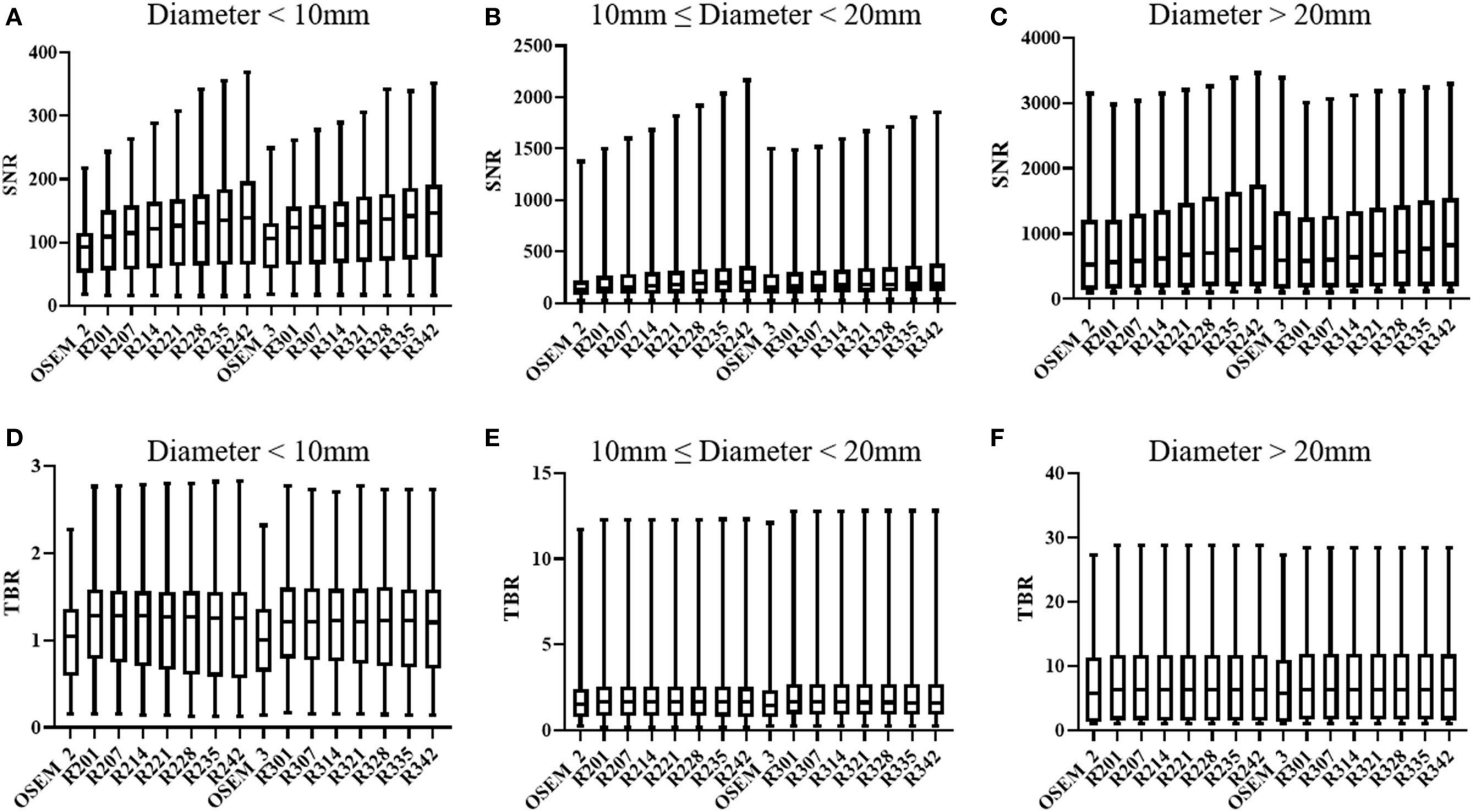
SNR and TBR based on different size of lesions: diameter < 10 mm **(A,D)**, 10 mm ≤ diameter < 20 mm **(B,E)**, diameter ≥ 20 mm **(C,F)**. The quartiles are represented by the bottom and top line, respectively. Median is middle line. SNR, signal-to-noise ratio; TBR, tumor-to-background ratio.

Figure 1 shows the results of lesions SNR and TBR. When lesions were divided into different groups according to their sizes, the SNR of small lesions (<10 mm) in most TVREM groups for 2 min/bed were higher than OSEM_3 significantly (all *p* < 0.05) except for R201 (*p* = 0.25), and the TBR of small lesions (<10 mm) in all TVREM groups were higher than OSEM significantly (all *p* < 0.05).

For medium lesions (≥ 10 mm but <20 mm), there were not significantly differences in the SNR between most TVREM groups and OSEM_3 except for R235 (*p* = 0.02) and R242 (*p* = 0.01), and the SNR of R214 was considered comparable to OSEM_3. The TBR of lesions in TVREM were higher than OSEM_3 significantly (all *p* < 0.05) except for R228 (*p* = 0.05), R235 (*p* = 0.06), and R242 (*p* = 0.07).

For large lesions (≥20 mm), there were not significantly differences in the SNR and TBR between TVREM groups and OSEM_3 (all *p* > 0.05).

**Figure 2:**
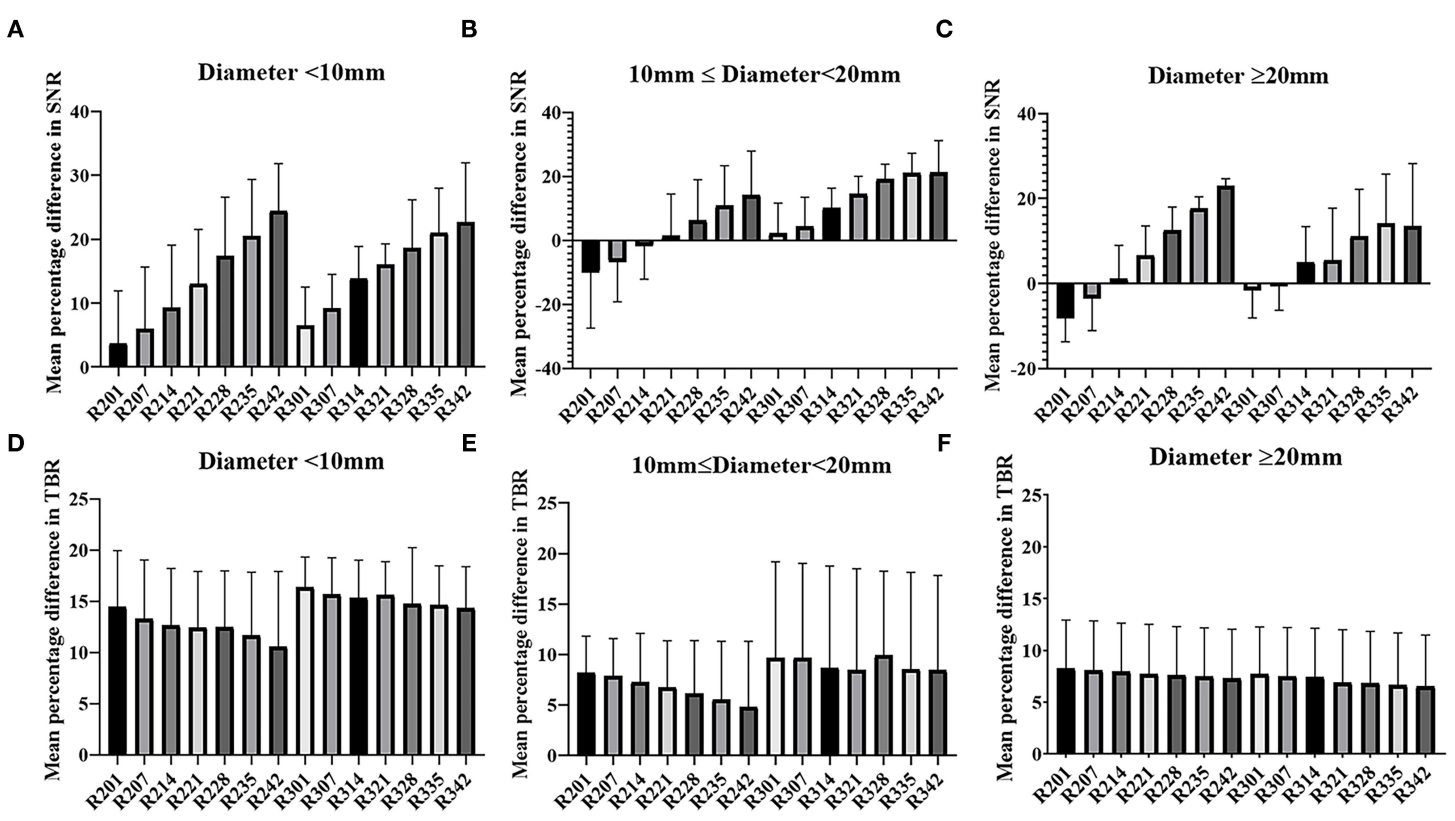
Mean and SD (error line) of the percentage difference in SNR and TBR of TVREM compared to OSEM_3 according to lesion size: diameter <10 mm **(A,D)**, 10 mm ≤ diameter <20 mm **(B,E)**, diameter ≥20 mm **(C,F)**. SD, standard deviation; SNR, signal-to-noise ratio; TBR, tumor-to-background ratio; TVREM, total variation regularized expectation maximization; OSEM, ordered subset expectation maximization.

**Figure 3:**
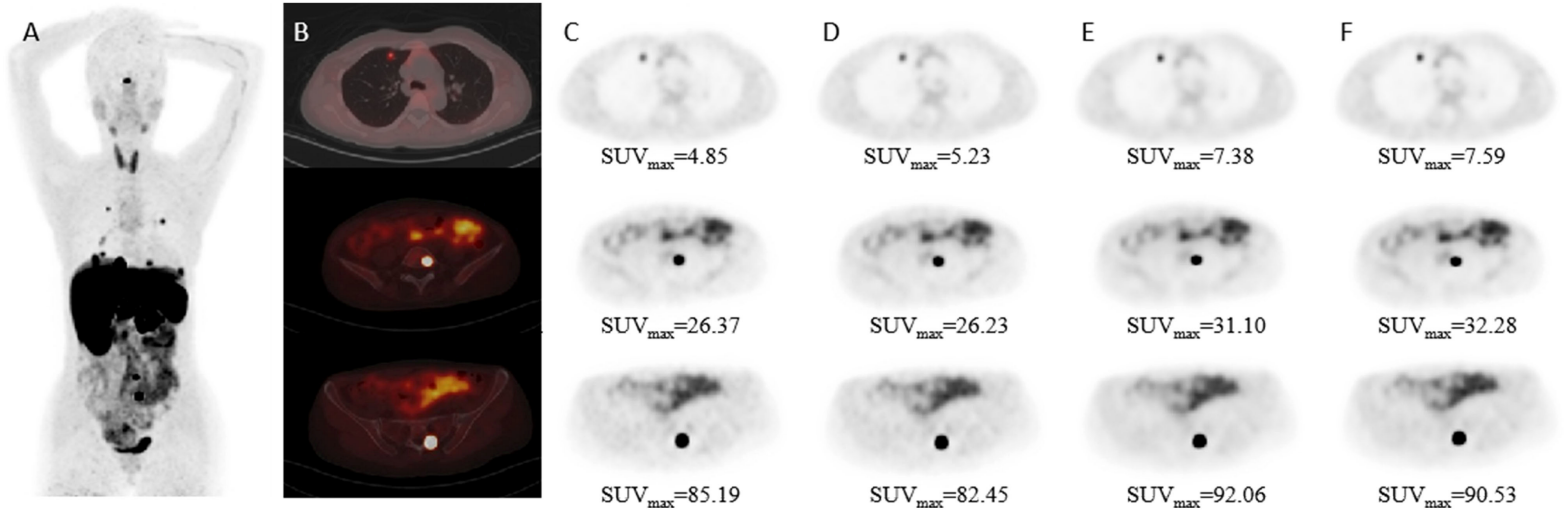
Patient images reconstructed by different algorithms. Patient with Adrenal pheochromocytoma (female; 44-years-old). **(A)** Maximum-intensity-projection ^68^Ga-DOTATAE PET shows multiple metastases. **(B)** Fused PET/CT images. **(C–F)** PET images with different size lesions in OSEM_2, OSEM_3, R221, and R314 groups. The diameter of the lesion was 8.40 mm in the first row, 16.86 mm in the second row, and 25.00 mm in the third row.

The mean percentage difference in the SNR and TBR between TVREM with difference penalization factors and OSEM_3 were shown in Figure 2. The mean percentage difference in the SNR of R242 was 19.25 ± 22.76% for small lesions, 12.79 ± 18.40% for medium lesions, and 18.13%± 9.80% for large lesions compared to OSEM_3. The mean percentage difference in the TBR of R201 was 14.31 ± 9.66% for small lesions, 8.06 ± 8.65% for medium lesions, and 8.88 ± 7.41% for large lesions compared to OSEM_3. A representative case for lesions with different sizes is shown in Figure 3.

**Figure 4:**
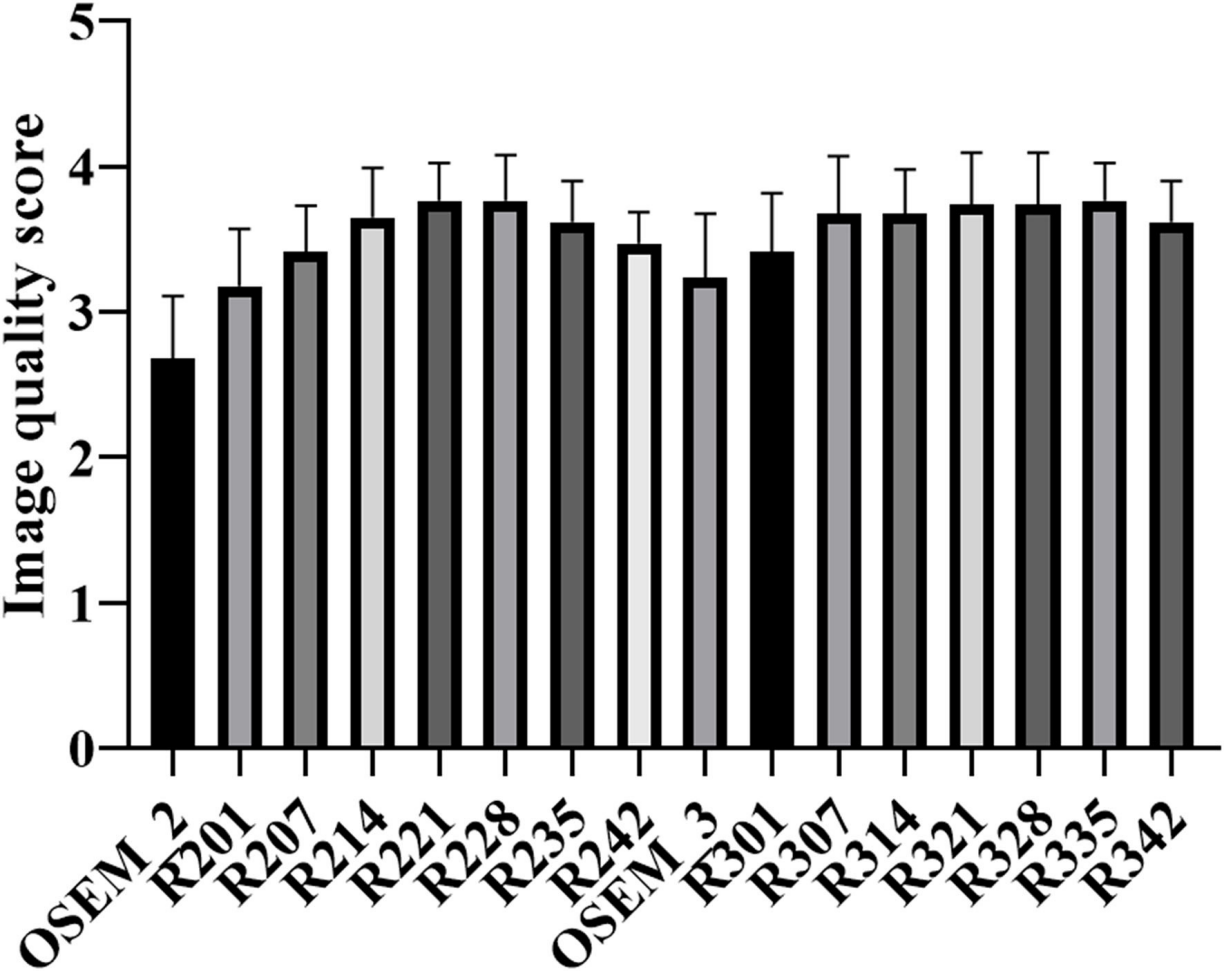
The mean and standard deviation (error bar) of the image quality score for TVREM and OSEM groups. The highest score was given to R221 and R335 for 2 and 3 min/bed acquisition. TVREM, total variation regularized expectation maximization; OSEM, ordered subset expectation maximization.

The results of subject evaluation for image quality were shown in Figure 4. The image quality scores of all the TVREM groups were higher than OSEM groups. R221 and R228 had higher image quality scores compared to OSEM_3 (*p* = 0.010 and 0.006), and the image quality scores of R328, R335, and R342 were significantly higher than OSEM_3 (*p* = 0.019, 0.004 and 0.029). The highest score was given to R221 (3.77 ± 0.26) in 2 min/bed groups and R335 (3.77 ± 0.26) in 3 min/bed groups. In all reconstruction series, the Kappa value of 0.45 indicated moderate reliability.

## Discussion

In our study, we evaluated the impact of TVREM on the image quality of ^68^Ga-DOTA-TATE. There was no significant difference in the liver SUV_mean_ between TVREM and OSEM, and TVREM showed a significant improvement of SNR and TBR compared to OSEM, especially for small lesions with diameter <10 mm. Moreover, we found that SUV_max_ values in TVREM were higher than that in OSEM significantly. TVREM with a penalization factor of 0.14 and 2 min/bed acquisition time could provide equivalent image quality compared to OSEM with 3 min/bed acquisition time.

It has been shown that BPL provided better image quality without affecting the background liver uptake and improved the lesions conspicuity (12, 13). Yoshie et al. showed that BPL reconstruction significantly improved the detection of small inconspicuous malignant tumors (diameter <10 mm) in the lung and improved the diagnostic performance of PET/CT (14). In our study, the results showed that the SNR and TBR of small lesions (diameter <10 mm) increased more than 3 and 6% in all TVREM groups compared to OSEM 3 min/bed. Those results were partly consistent with a previous study by Ewa et al. in which showed that BPL SUVs and TBR tend to be higher for small lesions (9). In addition, the SUV_max_ of lesions in our study decreased with the increase of penalization factor, which was consistent with a previous study using ^18^F-FDG PET/CT (15).

The capability of BPL algorithm of shortening acquisition time was reported in several studies (16, 17). A phantom and clinical study by Yang et al. showed that TVREM could improve the lesion contrast and lower image noise of ^68^Ga-PSMA-11 PET/CT compared to OSEM and enable a fast acquisition with 2 min/bed with preserved image quality (10). Similarly, the results in our study showed that a penalization factor between 0.21 and 0.42 for TVREM with 2 min/bed acquisition time could attain equivalent or lower noise compared to OSEM with 3 min/bed acquisition time.

New image reconstruction methods could help in clinical practice by reducing acquisition times while maintaining accuracy (18), getting better image quality in obese patients (19), improving accurately identify of very small lesions (20), and underpinning the use of further new tools such as data driven gating which can help to differentiate malignant lesions from begin structures and injuries (21). And the most accurate reconstruction factor were likely depending on the count statistics and uptake pattern vary between the radionuclide and radiopharmaceutical (22). In our subjective evaluation, the image quality was assessed using a 5-grade scale by two radiologists. Our results showed that the higher quality score was given to TRVEM groups with the penalization factor ranged from 0.14 to 0.35 for 2 and 3 min/bed acquisition. In our study, the image noise and TBR decreased with the increase of penalization factor. Although the increase of the penalization factor could reduce more noise, it could also reduce the contrast of the lesion, which was reflected in the lower image quality scores of the high-level penalization factor groups.

Our study has some limitations. First, the patient population was relatively small; a larger and multicenter study should be involved in the future. Second, the experience of two radiologists who evaluated the images were different, and the agreement was not very high. Furthermore, there was no pathologic confirmation of most lesions. The biopsy results should be used to investigate whether the TVREM could improve the detection rate of lesion in further studies.

## Conclusion

TVREM reconstruction algorithm can improve the SUV_max_, SNR and TBR and lower image noise of ^68^Ga-DOTA-TATE compared to OSEM, especially for small lesions <10 mm in diameter. TVREM has the potential to preserve image quality in short acquisition time with penalization factors ranged from 0.14 to 0.35.

## Data Availability Statement

The raw data supporting the conclusions of this article will be made available by the authors, without undue reservation.

## Ethics Statement

The studies involving human participants were reviewed and approved by the Affiliated Hospital of Southwest Medical University. The patients/participants provided their written informed consent to participate in this study.

## Author Contributions

LL conducted statistical analyses. LL and HL drafted the manuscript. SX, SZ, and YT collected cases. GM and YC provided critical review of the manuscript for key intellectual content. YC is the guarantor and as such, had full access to the data, and takes responsibility for its integrity and accuracy. All authors conceived and designed the study, interpreted the findings, and approved the final manuscript.

## Conflict of Interest

SX is employed by Shanghai United Imaging Healthcare Co., Ltd. The remaining authors declare that the research was conducted in the absence of any commercial or financial relationships that could be construed as a potential conflict of interest.

## Publisher's Note

All claims expressed in this article are solely those of the authors and do not necessarily represent those of their affiliated organizations, or those of the publisher, the editors and the reviewers. Any product that may be evaluated in this article, or claim that may be made by its manufacturer, is not guaranteed or endorsed by the publisher.
